# Estimating Life Expectancy in the U.S.: Identifying Environmental Determinants and Disparities (2003 to 2019)

**DOI:** 10.3390/s26206478

**Published:** 2026-10-13

**Authors:** Shisir Ruwali, David Lary, Samyak Shrestha, Faiz Ahmad

**Affiliations:** Department of Physics, The University of Texas at Dallas, Richardson, TX 75080, USA; shisir.ruwali@utdallas.edu (S.R.); samyak.shrestha@utdallas.edu (S.S.); faiz.ahmad@utdallas.edu (F.A.)

**Keywords:** life expectancy, machine learning, environment, particulate matter, formaldehyde, aerosols

## Abstract

We processed life expectancy data of an age group less than 1 years old from the Institute for Health Metrics and Evaluation (IHME) in the contiguous USA, as well as a set of 33 environmental variables (or features) from the European Center for Medium-Range Weather Forecasts (ECMWF) from the years 2003 through 2019. Visualizing the IHME data, we identified a massive disparity in life expectancy of the age group less than 1 years old in the contiguous USA, where counties in southern states have relatively lower life expectancies compared to counties in northern states. Our use of machine learning to estimate the life expectancy of the sub-1-year-old age group is moderately accurate, as the coefficient of determination (R^2^) and Root Mean Square Error (RMSE) between the true and estimated values was found to be 0.81 and 1.06 years, respectively, in an independent test set using only a set of five environmental variables. Our key finding shows that apart from well-known pollutants, such as particulate matter (PM), ozone, and carbon monoxide, a reduction in pollutants such as formaldehyde, sulphate aerosols, and dust aerosols; an increase in vegetation areas; and good environmental conditions, such as lower wet-bulb temperature, could potentially increase life expectancy in the US. Future work may include socio-economic variables such as household income, poverty rate, and other relevant features to create a comprehensive set of variables to improve the results and individuals’ quality of life.

## 1. Introduction

The trend of life expectancy and the factors that affect it have been studied for several years. Despite the growth in the economy, massive spending on health care, and medical innovations, life expectancy in the United States, compared to other high-income countries, falls behind [1]. Life expectancy in the U.S. increased between 1959 and 2016 from 69.9 years to 78.9 years, but it was on a decreasing trend after 2014 for three years [2,3]. The life expectancy of the total population at birth in the US was also found to be 79.0 years in the year 2024, which is an increase of 0.6 year compared to 2023 [4]. Previous studies have also described the disparity in life expectancy between various ethnic groups in the United States [5,6,7].

The human body interacts with the surroundings and is affected by several factors such as temperature, humidity, altitude, and air pollution. This, in turn, affects life expectancy. A decrease in the concentration of ${\mathrm{PM}}_{2.5}$ has been known to increase life expectancy [8,9]. Cohort studies also show that improved air quality results in good health, leading to a better life expectancy [10,11]. The temperature of the environment significantly affects health. High and low temperatures have been associated with years of life lost, as shown by data from seven locations with low-, middle-, and high-income sites [12]. An increase in the average annual temperature due to climate change could also lead to a decrease in life expectancy [13]. Health hazards are created not only by temperature conditions but also by humidity. Lower levels of humidity create a favorable condition for the transmission and spread of airborne diseases such as influenza [14,15]. Higher humidity makes it harder for the body to sweat, reducing its ability to cool itself.

Formaldehyde is a chemical that is used in building materials and in the production of several household products. Acute and chronic exposure to formaldehyde can have harmful effects on the respiratory system, urinary system, acute poisoning, irritation, and chest pain [16,17]. Environmental greenness is also a crucial factor affecting life expectancy. Greenness is known to affect human health by possibly encouraging physical activity [18], promoting mental health [19], and is beneficial in reducing air pollution [20].

In this study, we use data from two different sources: first, life expectancy data from the Institute for Health Metrics and Evaluation (IHME) [21], which provides county-wise life expectancy data from 2001 to 2019 for various age groups. We then consider a set of environmental variables from the European Center for Medium-Range Weather Forecasts (ECMWF). As we initially do not know which features are most influential in estimating life expectancy and the importance of these features, we gathered a comprehensive set of environmental variables from ECMWF that could possibly affect life expectancy in the U.S. Using these data sources, our study aims to achieve the following three objectives:

- Identify the disparity in life expectancy of the sub-1-year-old age group in the contiguous U.S., considering 17 years of county-wise life expectancy data from 2003 through 2019.
- Estimate life expectancy of the sub-1-year-old age group in the contiguous US using a set of environmental variables.
- Identify environmental factors that affect the life expectancy of the sub-1-year-old age group.

Rather than focusing on specific variables, such as temperature, humidity, and air pollution, our study considers a comprehensive set of environmental variables that could affect life expectancy. Furthermore, instead of a localized region and a small period of time, our study considers 17 years of county-wise data for the entire contiguous U.S. We also utilize machine learning to test the accuracy in estimating life expectancy of the sub-1-year-old age group in the U.S. using a set of environmental variables. We have also taken a machine learning approach to identify environmental factors that affect the life expectancy of the same age group in contiguous U.S.

## 2. Methodology

The methodology we implement first involves gathering life expectancy data for the sub-1-year-old age group from IHME and a set of environmental variables from ECMWF. We then match the temporal and spatial resolution of the environmental variables to our target variable, which is life expectancy for the sub-1-year-old age group. We also construct new features based on other features, and finally, we develop our machine learning model. A brief description of data sources is given below:

### 2.1. IHME Data

The University of Washington houses an independent global health research center called the Institute for Health Metrics and Evaluation (IHME) [21,22]. The IHME creates and supports a data catalog called the Global Health Data Exchange (GHDx). Improving the health of the world’s population is a mission of IHME, supported by GHDx by providing information related to population health. IHME provides a collection of datasets, among which we use the United States Mortality Rates by Causes of Death and Life Expectancy by County, Race, and Ethnicity 2000–2019 (https://ghdx.healthdata.org/record/ihme-data/united-states-causes-death-life-expectancy-by-county-race-ethnicity-2000-2019, accessed on 22 December 2025). Among the several csv files, we use the “life expectancy estimates 2000–2019, both sexes”. This life expectancy was estimated by IHME using the population and deaths data provided by the National Center for Health Statistics [22]. This csv file provides an estimate of life expectancy in the U.S. at the county, state, and national levels for each of the following:

- Age group: <1 year, 1 to 4, 5 to 9, 10 to 14, 15 to 19, 20 to 24, 25 to 29, 30 to 34, 35 to 39, 40 to 44, 45 to 49, 60 to 54, 55 to 59, 60 to 64, 65 to 69, 70 to 74, 75 to 79, 80 to 84, 85+.
- Racial-ethnic group: total population, Hispanic or Latino (Latino), non-Hispanic Black (Black), non-Hispanic White (White), non-Hispanic American Indian Alaska Native, Asian or Pacific Islander (Asian).

Among the three different geographic regions, various age and racial ethnic groups, we consider the life expectancy in counties for the age group less than 1 year of the entire population instead of a particular ethnic group.

### 2.2. Environmental Variables from ECMWF

The European Union’s Space program has an Earth observation component called Copernicus. ECMWF is a research institute that plays a key role in Copernicus by producing and disseminating weather data. The Copernicus Atmosphere Monitoring Service (CAMS) [23] and the Climate Change Service (C3S) [24] are two of six services provided by Copernicus and implemented by the ECMWF. We gather a set of environmental variables from CAMS and C3S. Data processed by ECMWF are gathered from approximately 90 satellite instruments, as well as ground and aircraft based sensors [25]. The services provided by Copernicus relies on the processing of extensive environmental data gathered from Earth observation satellites and in situ sensors. These in situ sensors comprise a wide range of sources such as air-quality monitoring networks, ground-based weather stations, and ocean buoys that provide reliable and integrated information [26]. The observations made by satellites are also used by CAMS to predict the emissions caused by wildfires and the use of fossil fuel. These satellite observations are also used to monitor the movement of gases such as ${\mathrm{CO}}_{2}$, methane (CH_4_), and nitrous oxide (N_2_O) between the atmosphere and surface of the Earth [27].

A brief description of the datasets is given below:

#### 2.2.1. CAMS Data

CAMS is a research institute that delivers reliable global data related to air pollution and health, greenhouse gases, solar energy, and climate forcing. Among the various datasets provided by CAMS, we use two of the datasets. The first is the CAMS global reanalysis (EAC4) (https://ads.atmosphere.copernicus.eu/datasets/cams-global-reanalysis-eac4?tab=overview, accessed on 22 December 2025), which stands for the ECMWF Atmospheric Composition Reanalysis 4. EAC4 is the fourth-generation global reanalysis of atmospheric composition produced by ECMWF. In the reanalysis procedure, the model data are combined with observations from all over the world. The second dataset that we use is the CAMS global reanalysis (EAC4) monthly averaged fields (https://ads.atmosphere.copernicus.eu/datasets/cams-global-reanalysis-eac4-monthly?tab=overview, accessed on 22 December 2025).

The CAMS EAC4 and EAC4 monthly-averaged data are gridded data covering the whole world at a horizontal resolution of 0.75 ° × 0.75 °, and currently cover the years 2003 to 2024. The variables by EAC4 are available every 3 h, whereas the EAC4 monthly averaged is available every one month. Vertical coverage consists of data at the surface level, total column, various model levels (or vertical distance), and pressure levels. The total column refers to the total amount of a chosen variable within a column of air extending from the Earth’s surface up to the top of the atmosphere. Both datasets have variables available at different vertical regions of the atmosphere.

From CAMS EAC4 we take variables at the surface level and only one total column variable, which is the total column ozone. We selected surface-level variables because humans reside mostly at the Earth’s surface. We included total column ozone because of the large abundance of ozone in the ozone layer located in the stratosphere. Ultraviolet-B are rays in the wavelength band (280–315 nm) that can reach the surface of Earth causing sunburns, DNA damage, and some forms of cancer as well [28]. This ozone layer is essential because it absorbs ultraviolet radiation, protecting living beings from harmful rays. From the CAMS EAC4 monthly averaged data, we take variables at the surface level. These variables were downloaded from their corresponding website using their Graphical User Interface (GUI). To obtain surface-level values, we selected model level 60 in the GUI. Some of the variables available from the CAMS EAC4 data are also available in the CAMS EAC4 monthly averaged, whereas some variables exist only in the CAMS EAC4 monthly averaged.

The list of variables that are used is given in Table 1 below. In Table 1, variables from 1 to 11, except total column ozone, are available only at the surface level. Variables 12 to 28 are multilevel variables; that is, they are available at various vertical distances in the atmosphere, but we choose the values at the surface level. Variables 29 to 32 are available only as monthly averages. Variables 12 to 27 have units ${\mathrm{kgkg}}^{-1}$ to indicate that the quantity is expressed as the mass mixing ratio; that is, the amount of the variable in a mixture compared to the total amount of all other variables. The Leaf area index high vegetation includes portions of land covered by vegetation of tall heights, such as evergreen trees, mixed forest/woodland, deciduous trees, and interrupted forest. The leaf area index low vegetation includes land covered with vegetation of small heights, such as crops and mixed farming, irrigated crops, tundra, short and long grass, semidesert, marshes and bogs, evergreen and deciduous shrubs, and land and water mixtures. An index of 0 indicates bare land.

**Figure 1:**
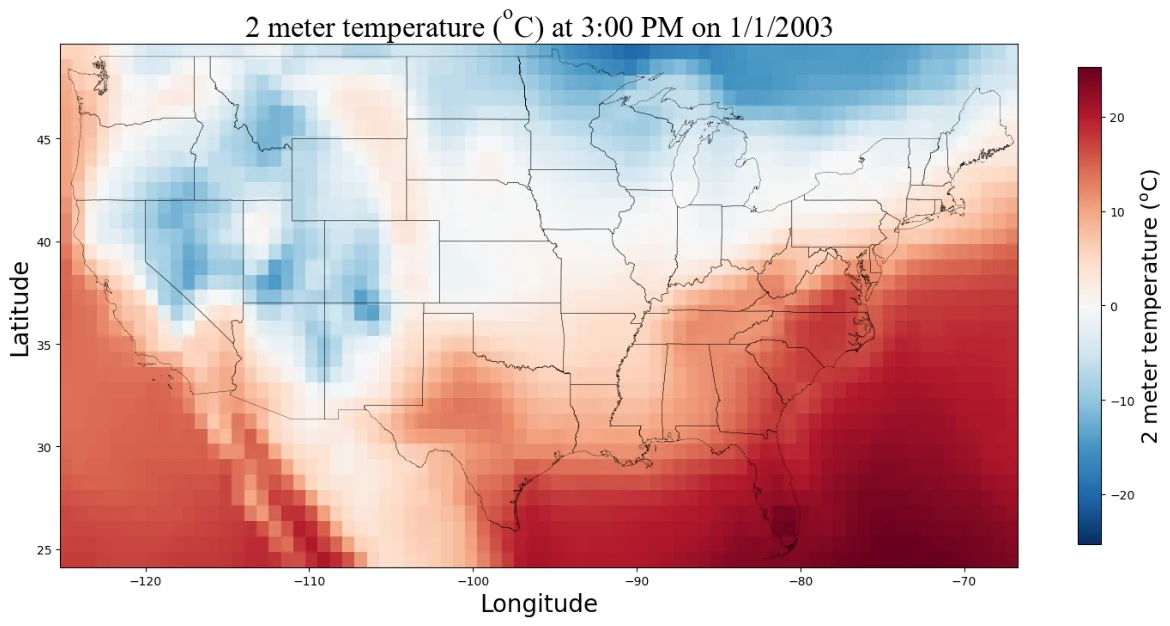
Two-meter temperature (°C) at 3:00 p.m. on 1 January 2023 with the boundary of contiguous states overlaid. Data source: CAMS EAC4 [23].

Among the list of variables, Figure 1 shows the 2-m temperature in degrees Celsius at 3:00 p.m. on 1 January 2023 within the given latitude and longitude box. The boundary of the states of the contiguous U.S. is overlaid with black boundaries. The resolution is 0.75 ° × 0.75 ° because of which the image looks grainy. The image shows that the southern region was warmer than the northern region during the given time. Most of the northwest region was cooler than other regions as well.

#### 2.2.2. ERA5 Data

**Figure 2:**
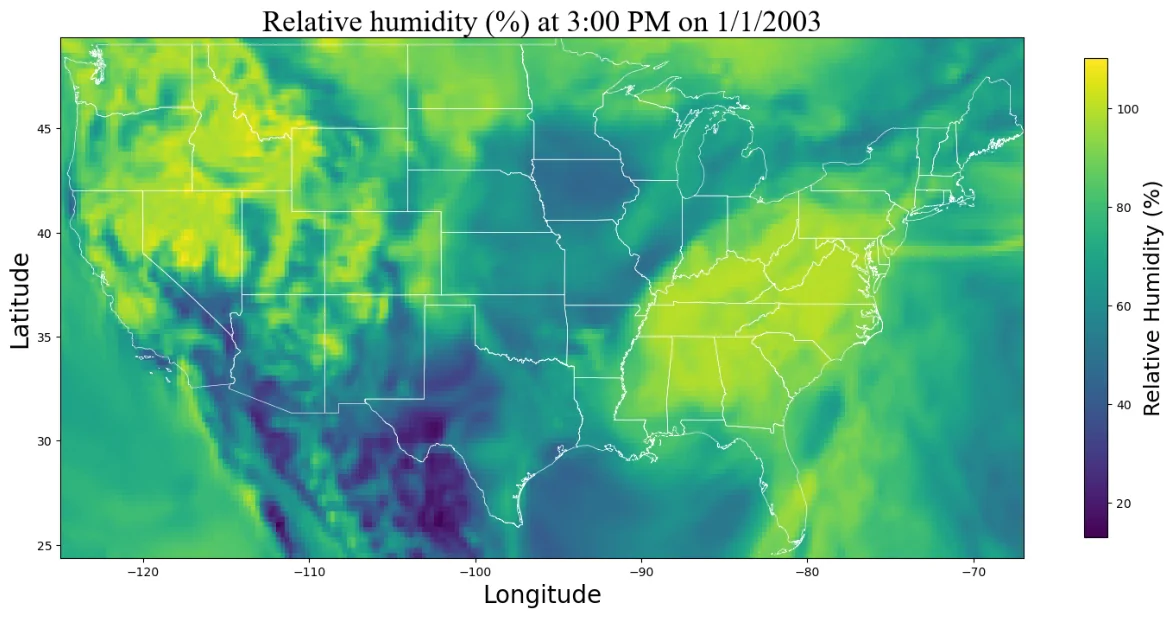
A timestamp of Relative humidity (%) at 3:00 p.m. on 1 January 2023 with the boundary of contiguous states overlaid. Data source: ERA5 [24].

The ERA5 data are available from 1940 to the present day. The variables are available every hour but were downloaded with a resolution of every 3 h because of the limitation on the amount of data that can be downloaded at a single time. Among the variables available from ERA5, we consider relative humidity as it was not available in CAMS EAC4. Figure 2 shows a timestamp of relative humidity data at 3:00 p.m. on 1 January 2023. The resolution of the data is 0.25 ° × 0.25 °, which makes the image look smoother. The figure clearly shows higher relative humidity in the southern and western United States than in other regions at that timestamp.

### 2.3. Matching Temporal and Spatial Resolution

The data sources used in this study have different temporal and spatial resolutions. A summary of the resolutions is given in Table 2 below.

The target variable we consider is life expectancy data from IHME, so we match the temporal and spatial resolution of the rest of the data to the IHME data. The temporal and spatial resolution is resolved in the following way:

- Temporal resolution: The life expectancy data from IHME is annual. We calculate a one year average of each of the variables from CAMS EAC4, CAMS EAC4 monthly averaged, and ERA5. For example: the temporal resolution of the CAMS EAC4 data is 3 h, so 1 year has a total of (24/3) × 365 = 2960 data points. We calculate the average of these 2960 data points.
- Spatial resolution: The CAMS and ERA5 data are raster data, whereas the IHME data are at the county level. To resolve this, multilinear interpolation is performed, in which the raster data from each of the data sources is interpolated to the boundary points of the counties. These boundary points are accessed from the shapefile of the counties for a particular year. Shapefiles are a set of files that store the location, shape, and attributes of geographic features such as points, lines, and polygons. These shapefiles were downloaded from the United States Census Bureau website (https://www.census.gov/geographies/mapping-files/time-series/geo/carto-boundary-file.2010.html#list-tab-1556094155, accessed on 22 December 2025). The shapefiles for the years 2003 and 2007 could not be found, so for these years, shapefiles for 2008 are used. The pair of latitude and longitude points available for a county from a shapefile can vary. For counties that have more than 100 pairs of latitude and longitude available, multilinear interpolation of each of the variables was done on 100 pairs of latitude and longitude and then averaged. For counties where there were fewer than 100 latitude and longitude pairs available, multilinear interpolation of each of the variables is done to that many pairs of latitude and longitude and then averaged. The total number of counties in each year, the smallest number of pairs of latitude and longitude available among all counties, and the total number of counties where there were fewer than 100 pairs of latitude and longitude available for each year are given in Table A1 in Appendix A.

**Figure 3:**
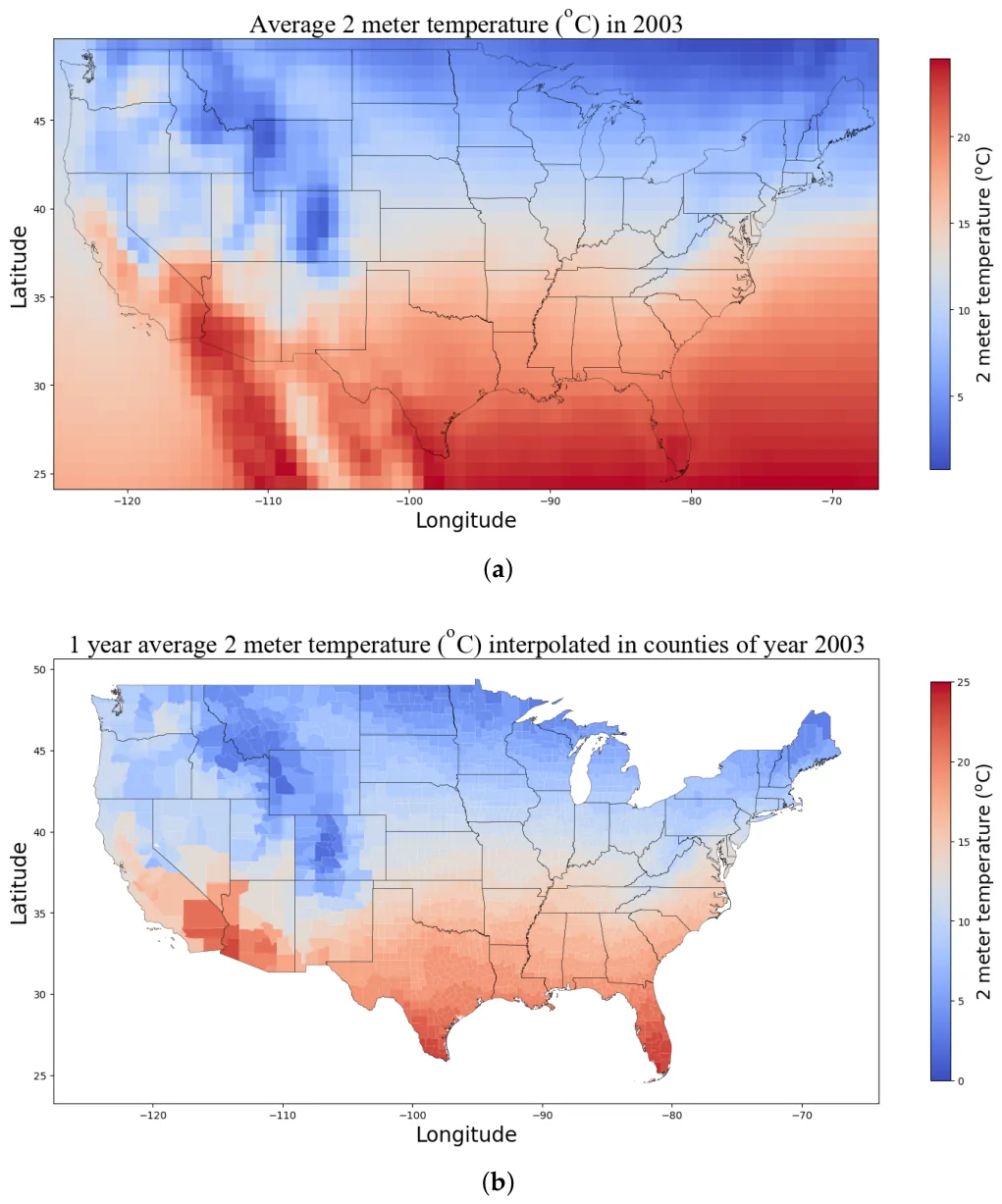
(**a**) Raster plot of the 1-year average of 2-m temperature for the year 2003, calculated using data from CAMS EAC4, with state boundaries overlaid. (**b**) Plot where multilinear interpolation was performed on boundary points of counties and then averaged to find corresponding values of the variable in each county, with boundaries of states overlaid.

Figure 3a shows the raster data where the average 2-m temperature for the year 2003 is calculated using the data from CAMS EAC4. Figure 3b shows the corresponding values in the counties after the raster data is multilinearly interpolated to 100 or less pairs of latitude and longitude pairs (as available from the shapefile) and then averaged. The calculated values in Figure 3a are almost identical to the interpolated values in Figure 3b, indicating an excellent representation of the variable at the county level.

### 2.4. Feature Engineering

The data from CAMS EAC4 and CAMS EAC4 monthly averaged (32 variables) and the relative humidity data from ERA5 make up a total of 33 variables. We also feature engineering (the process of constructing new features) using a subset of these 33 features. A set of two feature engineered variables is explained below:

- Wind velocity: The 10m u-component of wind and the 10m v-component of wind are the eastward and northward components of the wind at a height of 10 m, respectively. Using these two features, we feature engineer wind velocity using the following formula:
  (1) $$\;\mathrm{Wind}\;\mathrm{Velocity}=\sqrt{{\left(10\;m\;u-\mathrm{component}\;\mathrm{of}\;\;\mathrm{wind}\right)}^{2}+{\left(10\;m\;v-\mathrm{component}\;\mathrm{of}\;\mathrm{wind}\right)}^{2}}$$
  This process is done to reduce the number of features. Instead of using two different features: the 10m u-component of wind and 10m v-component of wind, we instead use wind velocity.
- Wet bulb temperature: Wet bulb temperature is measured by wrapping the bulb of a thermometer with a wet muslin. Measuring this temperature is important because it indicates how much a body can be cooled by evaporation. Dry air can absorb water faster than humid air. Lower levels of humidity leads to a lower wet bulb temperature, and our body can stay cooler through evaporation. However, if the relative humidity is 100%, the wet bulb temperature will be identical to the dry bulb since the water in the wet muslin cannot evaporate. The wet bulb temperature is calculated as follows [29]:
  (2) $$\begin{array}{c} \;\mathrm{Wet}\;\mathrm{bulb}\;\mathrm{temperature}=T\;\arctan[0.151977{\left(\mathrm{RH}\%+8.313659\right)}^{1/2}]+\arctan(T\;+\;\mathrm{RH}\%)\\ -\arctan\;(\mathrm{RH}\%-1.676331)\\ +0.00391838{\left(\mathrm{RH}\%\right)}^{3/2}\arctan\left(0.023101\mathrm{RH}\%\right)-4.686035] \end{array}$$
  where air temperature is expressed in °C and relative humidity is expressed in %. We calculate the 1 year average temperature and 1 year average relative humidity of a each year and used Equation (2) to calculate the wet bulb temperature for that particular year.

Replacing the 10m u-component of wind and 10m v-component of wind with wind velocity and adding wet bulb temperature in the list of features makes up a total of 33 features.

We also feature engineer 12 additional variables to consider extreme cases such as high temperature, low temperature, and fraction of time (FoT) where a county exceeded the threshold of the ${\mathrm{PM}}_{2.5}$ standard set by the Environmental Protection Agency (EPA). Exposure to heat can affect the regulation of internal temperature in the human body [30]. A study has shown that extremely high temperatures were associated with human mortality during the 2010s in the United States [31]. Short-term exposure to extreme cold temperatures below 5 °C can lead to cold stress, drastic physiological changes such as changes in heart rate and blood pressure, and affect the ability to make judgments [32]. Health effects also tend to be prominent among elderly people in high-temperature conditions compared to low-temperature conditions [33]. The list of 12 features that are engineered is given in Table 3.

Among the features shown in Table 3, for features 11 and 12, the corresponding threshold was set, and the number of times a county was above the threshold is calculated from the 2920 timestamps of the data provided by CAMS EAC4 and then multiplied by 100. The threshold set by EPA for ${\mathrm{PM}}_{10}$ particles is 150 μ ${\mathrm{g/m}}^{3}$ for a 24-h average, and the threshold set by EPA for ${\mathrm{PM}}_{2.5}$ is 9.5 μ ${\mathrm{g/m}}^{3}$. For the rest of the variables, after the values of the variable in a county is calculated as mentioned in Section 2.3, the 90th or the 10th percentile was calculated among all counties in the particular year. From the set of 2960 timestamps as provided by CAMS EAC4, the number of times the variables were above the 90th percentile (or below the 10th percentile) multiplied by 100 is calculated.

With the set of 12 feature engineered variables, the total number of features we have considered is 45.

### 2.5. Machine Learning Model Development

Before using machine learning, we first test whether any subset of features is linearly correlated since we used a total of 45 features. The information carried by one of the features will be almost identical to the rest of the features with which it is highly linearly correlated, and we can choose a single feature from a group of multicollinear features, which will reduce the number of features. To identify the cluster (or groups) of features that are linearly correlated, we make use of a dendrogram. A dendrogram and further details are explained in Appendix B.

From the dendrogram, we can identify a set of nine clusters (or groups) of features that are multicollinear, which are shown in Table 4. As each of the features in the group is highly linearly correlated with each other, we can use any of the features, as they contain almost identical information. We use features that are easier to interpret among these set of features. The feature that is chosen is mentioned first in each of the groups.

The remaining set of 21 features are given in Table 5.

This provides us with a total of 30 features, among which 24 features are distributed in 9 different groups, and 21 features are not multicollinear with any other feature.

The final tabular dataset where we make use of machine learning consists of 31 columns, which include 30 features and 1 target variable (life expectancy), and 52,320 rows (or samples of data) from 17 years of life expectancy data. To estimate the life expectancy of the sub-1-year-old age group, we utilize the Random Forest algorithm [34] using scikit-learn [35] for non-linear, multidimensional data. As our dataset is tabular, tree-based models such as Random Forest tend to be more efficient than other models [36,37]. To benchmark the accuracy of the Random Forest model, we also use the XGBoost model. Moreover, 80% of the dataset is randomly chosen for training, and the remaining 20% of the dataset is used as an independent test set. To quantify the accuracy of the estimation, the root mean square error (RMSE), the coefficient of determination ($R^{2}$), and adjusted $R^{2}$ is calculated between the true and estimated values. The adjusted $R^{2}$ takes into account the number of features in a model, whereas the coefficient of determination does not. The adjusted $R^{2}$ is calculated using the following formula:

(3) $$1-\frac{\left(1-R^{2}\right)\left(n-1\right)}{n-k-1}$$

where

$R^{2}$ is the coefficient of determination between the true and estimated values
k is the total number of variables in the regression model
n is the number of samples in the data

To qualitatively assess the goodness of fit, we also plot a scatter plot and a quantile–quantile plot between the true and estimated values of life expectancy.

To identify the features that were the most important in estimating life expectancy, we make use of permutation feature importance and the SHAP (Shapley additive explanations) values [38,39] from the SHAP library. In permutation feature importance, a single feature is randomly shuffled, and the degradation of the model is observed. This process is done for every feature of the model. If the degradation is high after a feature is shuffled, the feature is considered to be of high importance, as there is high dependency between the feature and the target variable. On the other hand, if the degradation is low, then the feature is considered to be of low importance, as the feature has low dependency on the target variable. If any of the features is highly linearly correlated with any other feature, then when the feature is permuted, the model can still have access to the feature from the other feature with which it is linearly correlated. This is also one of the reasons for getting rid of multicollinearity among features using a dendrogram. Moreover, preparation of data and preprocessing steps has also been hypothesized to possibly mitigate underfitting as well as sampling error [40]. Further details about permutation feature importance are given in Appendix C. Permutation feature importance has a major limitation. “Permutation importance does not reflect to the intrinsic predictive value of a feature by itself but how important this feature is for a particular model” [41]. Due to this limitation, we also used SHAP values, which use a game theory approach to identify the importance of features.

## 3. Results

We first identify the disparity in life expectancy in the contiguous U.S. for the sub-1-year-old age group from the year 2003 to 2019. Then we estimate the life expectancy of the same group in the U.S. using a set of environmental factors and also identify the environmental factors that were most influential in affecting the life expectancy of the sub-1-year-old age group.

### 3.1. Identifying Disparity in Life Expectancy of Sub-1-Year-Old Age Group

We use county-wise life expectancy data for the sub-1-year-old age group, as provided by IHME, from the year 2003 through 2019. Table 6 shows the year, the corresponding county having the highest and lowest life expectancy for the sub-1-year-old age group, and the mean, median, and standard deviation for that year. For each year, the highest life expectancy for the sub-1-year-old age group was between two counties in Colorado, and the county with the lowest life expectancy for the sub-1-year-old age group was Oglala Lakota County in South Dakota. In each year, the difference between the highest and lowest life expectancy of the sub-1-year-old age group is greater than 20 years; the standard deviation is consistently over 2 years and even above 2.6 years in some cases, indicating the overall disparity in life expectancy of the sub-1-year-old age group.

**Figure 4:**
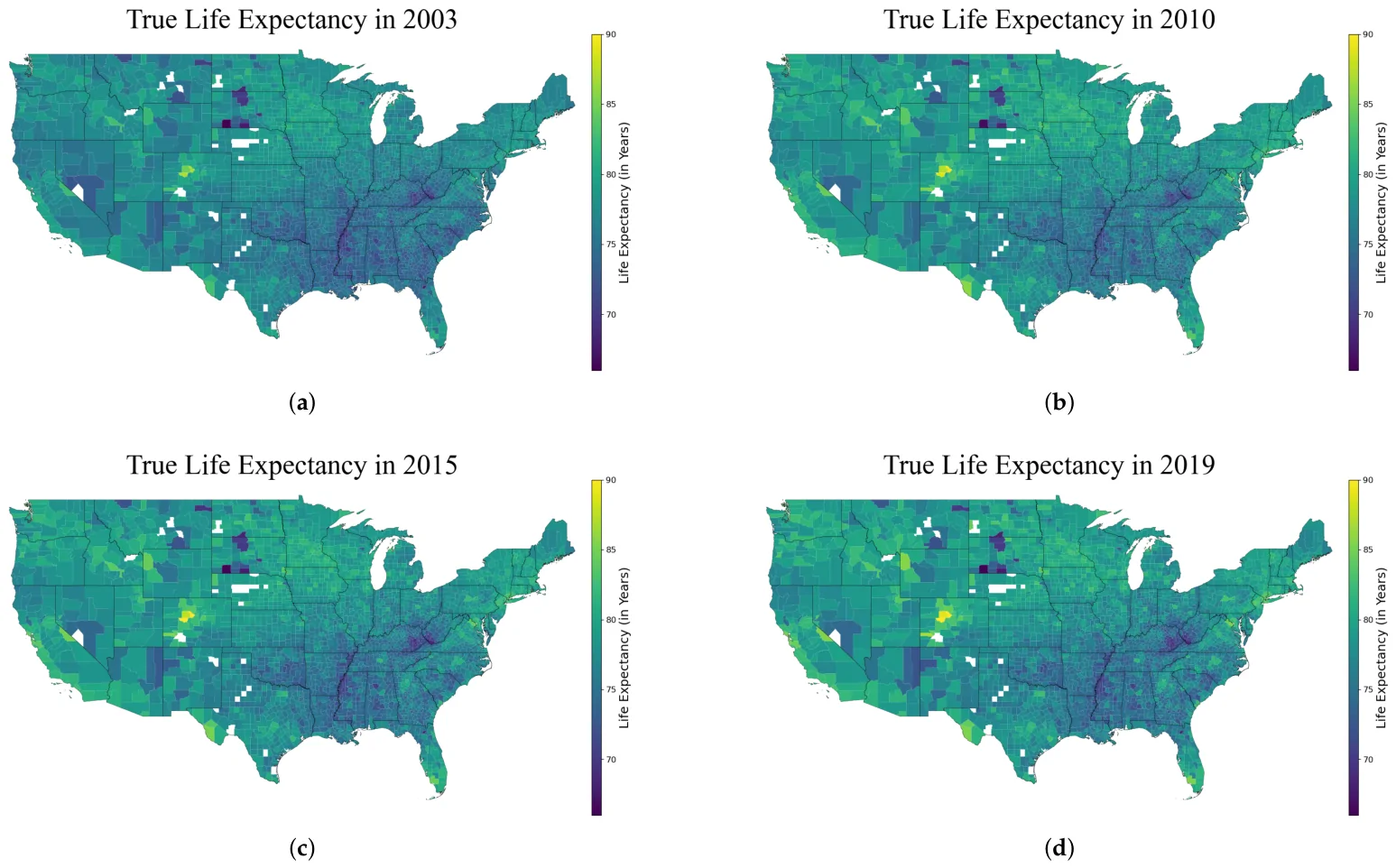
True life expectancy of the sub-1-year-old age group in the years (**a**) 2003, (**b**) 2010, (**c**) 2015, (**d**) 2019. White spaces indicate counties with no life expectancy data. The figure clearly indicates that southern regions have relatively lower life expectancy compared to northern states.

Figure 4 shows the life expectancy of the sub-1-year-old age group in the contiguous US for the years 2003, 2010, 2015, and 2019. White spaces indicate counties where life expectancy data do not exist. A gif file showing the life expectancy from 2003 to 2019 is available in the following link: (https://github.com/mi3nts/Life_Expectancy_Complete/blob/main/Figures/True_LE_plots/True_LE_gif.gif, accessed on 28 December 2025). A visual inspection of the gif file shows that the overall map appears to go from dark to light, indicating that the overall life expectancy of the sub-1-year-old age group increases over the years. However, a massive disparity in life expectancy is also observed in each year, where counties in southern states have relatively lower life expectancy compared to counties in northern states for the sub-1-year-old age group.

### 3.2. Estimating Life Expectancy of Sub-1-Year-Old Age Group in the U.S. Using Environmental Variables and Identifying Environmental Variables Affecting the Life Expectancy of the Same Age Group

We estimate the life expectancy of the sub-1-year-old age group in the US using a set of environmental variables and identify environmental factors that affect life expectancy. We first use a set of 30 features and then reduce the number of variables to test if the same accuracy can be obtained.

#### 3.2.1. Using 30 Features

**Figure 5:**
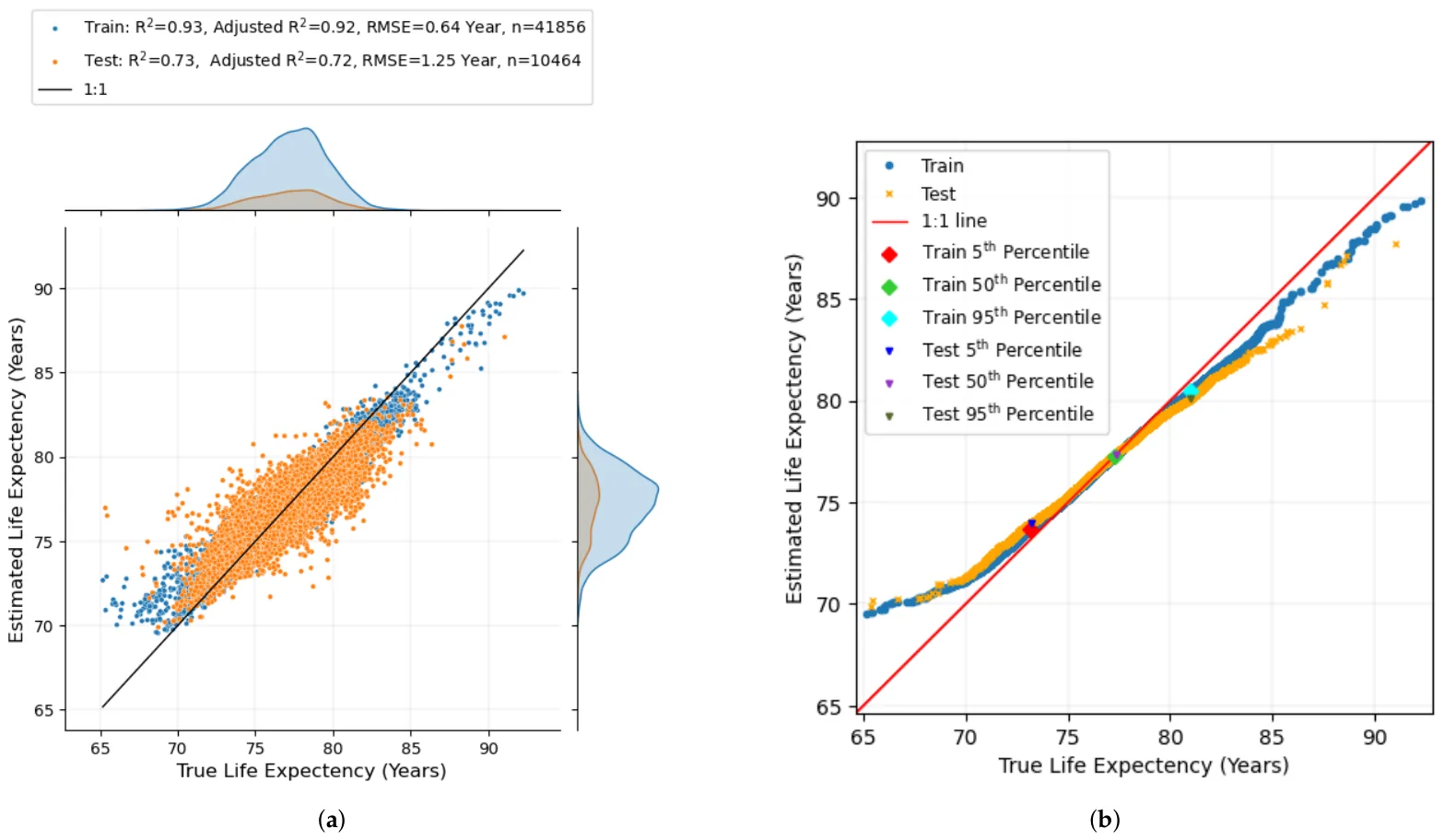
(**a**) Scatter plot of true values of life expectancy of the sub-1-year-old age group plotted against the estimated values of life expectancy of the sub-1-year-old age group when 30 features were used for estimating the life expectancy. A perfect fit is indicated by the black 1:1 line. (**b**) Quantile–quantile plot of true values of life expectancy of sub-1-year-old age group plotted against the estimated values of life expectancy of sub-1-year-old age group when 30 features were used for estimating life expectancy. A perfect fit is indicated by the red 1:1 line. The 5th, 50th, and 95th percentiles in each of the training and testing set is also overlaid.

We use a set of 30 features to estimate the life expectancy of the sub-1-year-old age group in the US using the random forest algorithm from scikit-learn with optimized hyperparameters as explained in Section 2.5. The list of hyperparameters that were optimized and the one selected is given in Table A2 in Appendix D. The results of the XGBoost model is found to be similar to the Random Forest model which can be found in Table A3 of Appendix D; we have, thus, used Random Forest in this study. As we randomly select 80% of the data for training and 20% of the data as an independent test set, we also use 10-fold cross-validation to test whether the selection of data makes a difference in our results where the training set itself is divided into 10 subsets, trained in 9 sets and tested in 1 set in a round-robin way. The result of 10-fold cross-validation is shown in Table A4 in Appendix D. Since the standard deviation of the RMSE of 10-fold cross-validation is found to be 0.02 years, this shows that our result does not change significantly when using a random selection of data. The list of 9 features used is given in Table 4, where we have used the first feature from each of the 9 groups of features, and the remaining 21 features are given in Table 5. The scatter plot and the quantile–quantile plot of estimation using 30 features are given in Figure 5a and Figure 5b, respectively. The quantified metric of goodness of fit is also shown on top of Figure 5a.

The result of the estimation is found to be moderate when we use 30 features. The $R^{2}$, adjusted $R^{2}$ and RMSE in the training set are found to be 0.93, 0.92 and 0.64 years, respectively. This high accuracy is expected, as this part of the data is used for training. The accuracy in the testing set is found to be moderate, as we obtain the $R^{2}$, adjusted $R^{2}$, and RMSE to be 0.73, 0.72, and 1.25 years, respectively.

Figure 5a shows the scatter plot of the estimation. The density curves on the top and right sides of the figure indicate that for a large number of counties from 2003 through 2019, the life expectancy of the sub-1-year-old age group was between 70 and 85. As we obtain the $R^{2}$ value of 0.93 and the RMSE of 0.64 years in the training set, the data points of this set are closer to the 1:1 line compared to that in the testing set, where we can observe that a large number of data points deviate from the perfect 1:1 line. Figure 5b shows the quantile–quantile plot of the estimation, where the quantiles of the true life expectancy of the sub-1-year-old age group and the quantiles of the estimated life expectancy of the sub-1-year-old age group are plotted on the X and Y axes, respectively. The 5th, 50th, and 95th percentiles of each distribution are also overlaid. The plot shows that the estimate was close to the perfect 1:1 line for data points between the 5th and 95th percentiles, whereas the estimate deviates from the 1:1 line below the 5th and above the 95th percentile in each of the training and testing sets.

**Figure 6:**
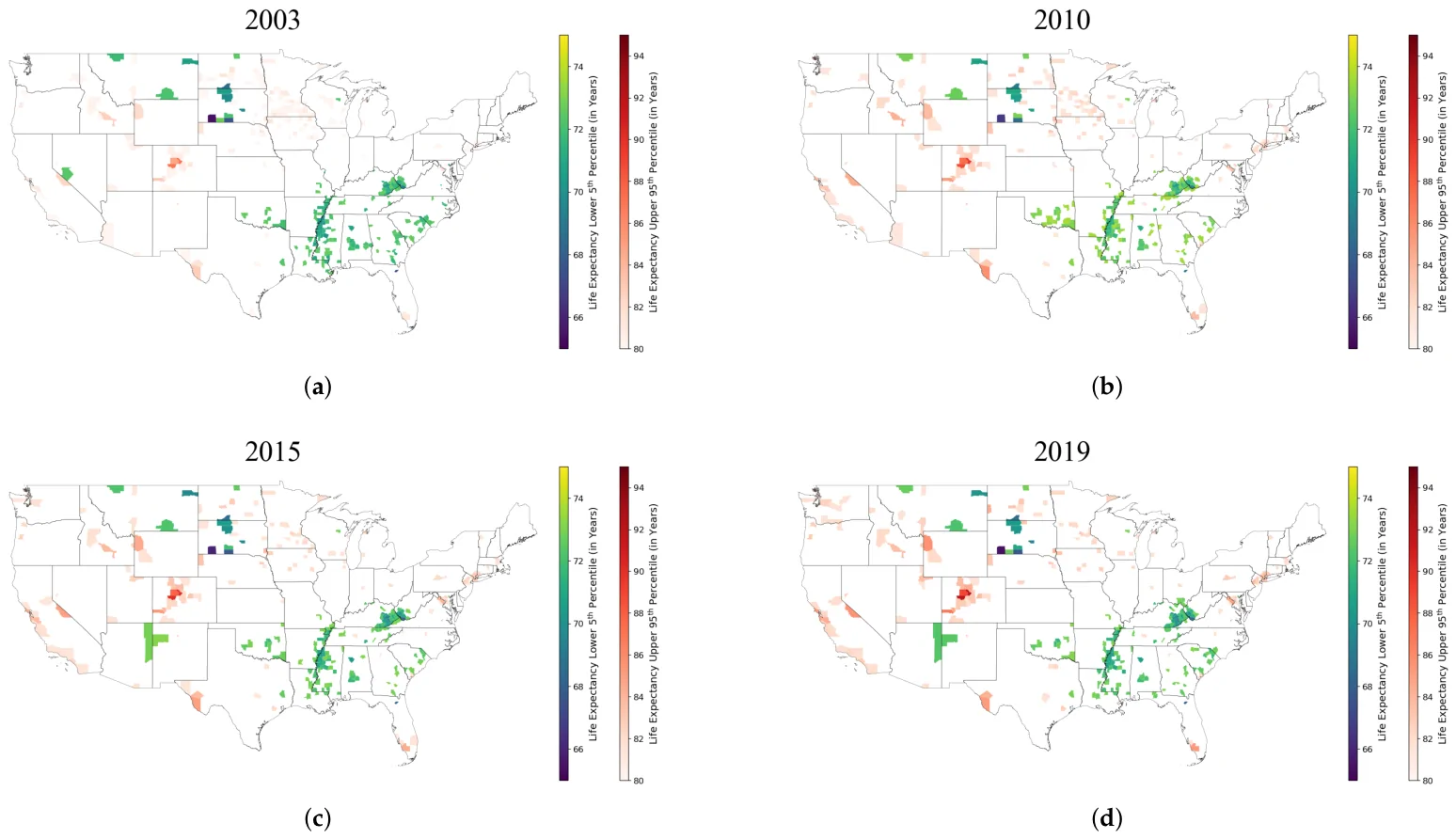
Life expectancy of sub-1-year-old age group in the year (**a**) 2003, (**b**) 2010, (**c**) 2015, and (**d**) 2019 where the counties below the 5th percentile are represented by color pallette on the left and above 95th percentiles are shown by the color palette on the right. The life expectancy below the 5th percentile is mostly clustered in the southern states, whereas above the 95th percentile, it is scattered throughout the contiguous US.

As the deviation from the perfect 1:1 line is below the 5th percentile and above the 95th percentile, we also visualize the life expectancy data for the sub-1-year-old age group below the 5th percentile and above the 95th percentile, which is shown in Figure 6.

The following link (https://github.com/mi3nts/Life_Expectancy_Complete/blob/main/Figures/Extreme_LE_plots/Extreme_LE_gif.gif, accessed on 28 December 2025) shows a gif file of images from the year 2009 through 2019 and the corresponding 5th percentile and the 95th percentile of the life expectancy of sub-1-year-old age group. Each of the figures shows that most of the counties with life expectancy below the 5th percentile are located in the southern states, whereas counties with life expectancy above the 95th percentile are scattered throughout the contiguous US.

**Figure 7:**
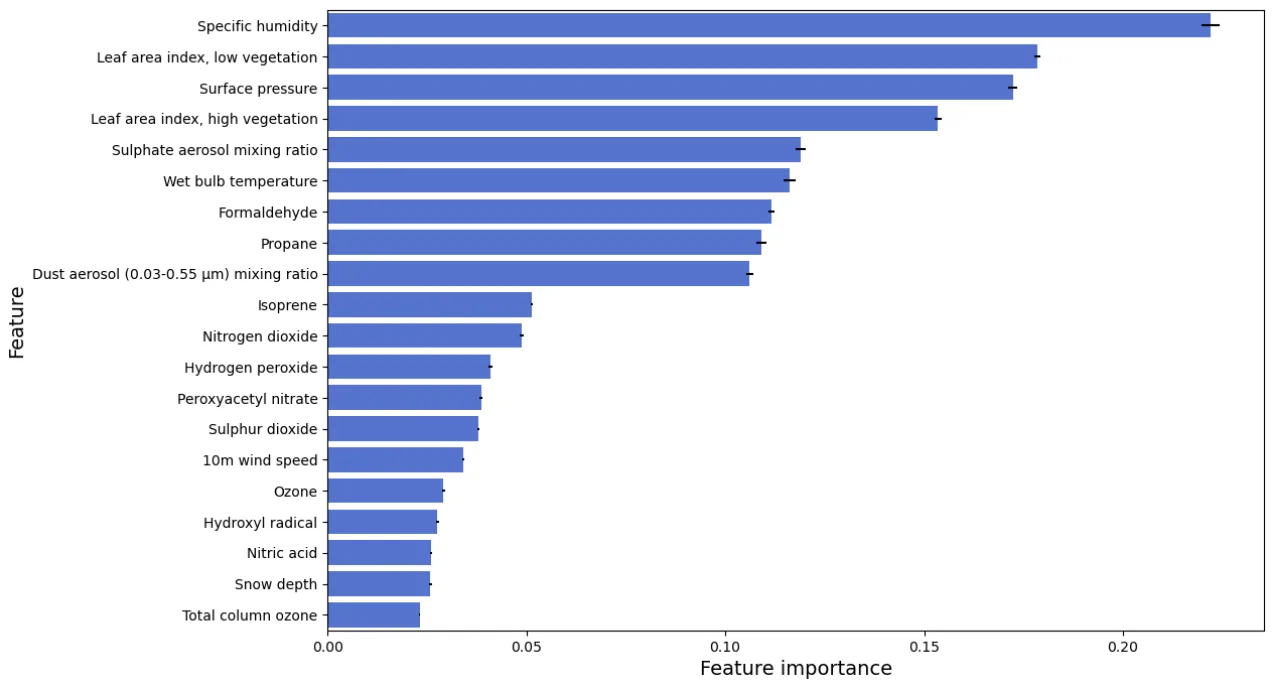
Feature ranking of variables that were most influential in estimating life expectancy for the sub-1-year-old age group, according to permutation feature importance in the training set when 30 features were used for estimation. The error bar represents the standard deviation of the variable when the feature was permuted 10 times, and the degradation of the model was calculated. Features are arranged in descending order, with the most influential features at the top. The top 20 features are shown.

In order to identify the importance of features that affect the life expectancy of the sub-1-year-old age group, we first used permutation feature importance. Figure 7 shows the feature ranking of 20 features in the training set. A complete feature ranking of 30 features is given in the link: (https://github.com/mi3nts/Life_Expectancy_Complete/blob/main/Results/permutation_importance_30_train.png, accessed on 28 December 2025). Each of the features is permuted 10 times, and the mean value of the degradation of the model is calculated. The features are arranged in descending order of the mean value, and the standard deviation is plotted as an error bar.

**Figure A2:**
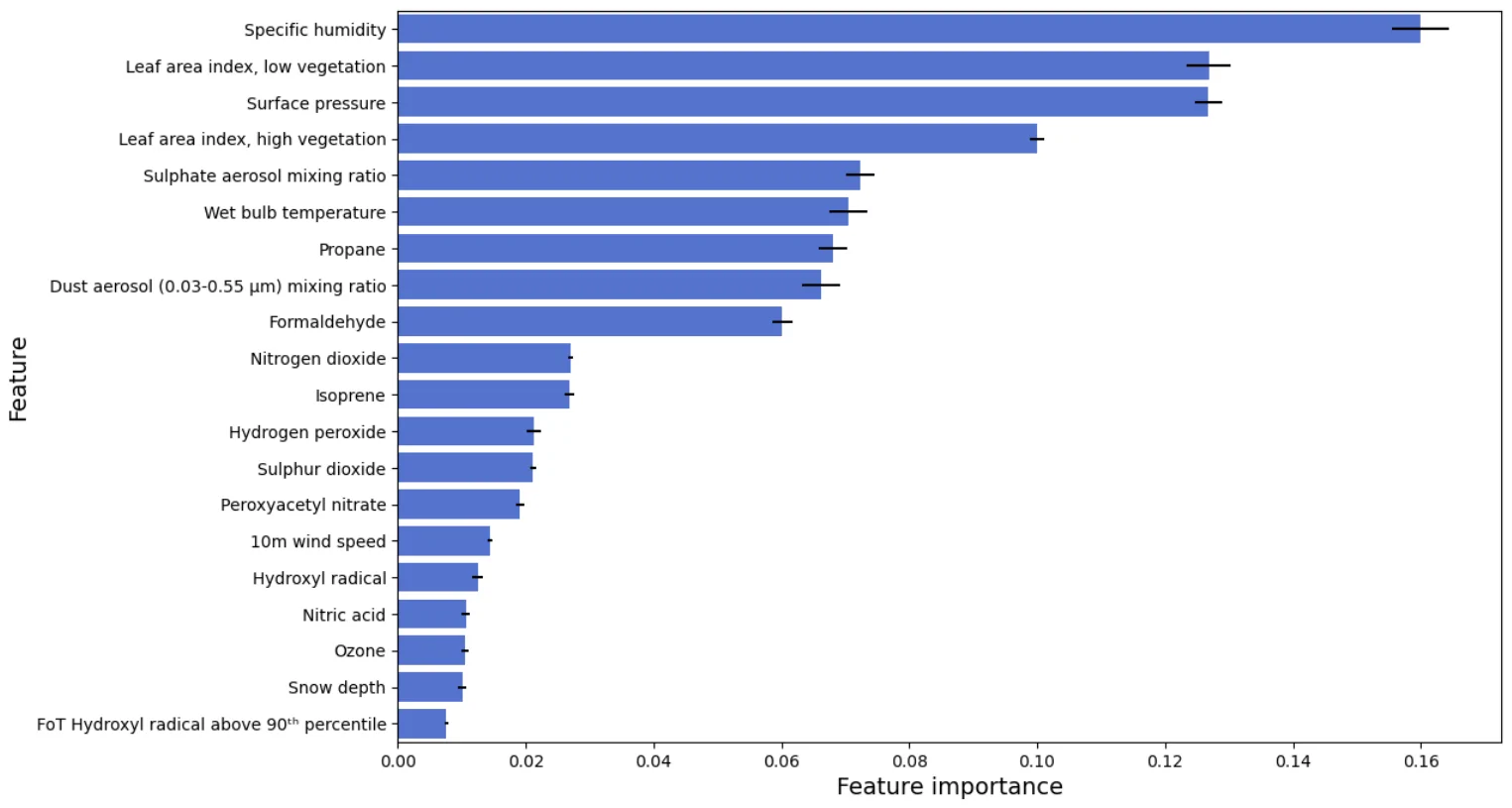
Feature ranking of top 20 variables using permutation feature importance in the test set in a descending order when 30 features were used for estimating life expectancy of sub-1-year-old age group. Each of the feature was shuffled 10 times and mean value is plotted. The standard deviation is plotted as error bar.

Figure 7 shows that leaf area index, low vegetation, and specific humidity are among the most influential features that affect the life expectancy of the sub-1-year-old age group. Other pollutants such as formaldehyde, sulphate aerosols, dust aerosols, and sulfur dioxide are among the most important features, while pollutants such as ${\mathrm{PM}}_{2.5}$, and ozone were in the bottom half of the ranking, indicating their relative importance is small compared to other variables at the top. As the standard deviation of each of the features is small, the ranking of the features will tend to be the same when the algorithm is rerun. As there are considerable differences in accuracy in estimating the life expectancy of the sub-1-year-old age group in the training and testing sets, a feature ranking is also conducted on the testing set. The permutation feature ranking in the testing set is given in Figure A2 of the Appendix E. The ranking in the testing set is almost identical.

**Figure 8:**
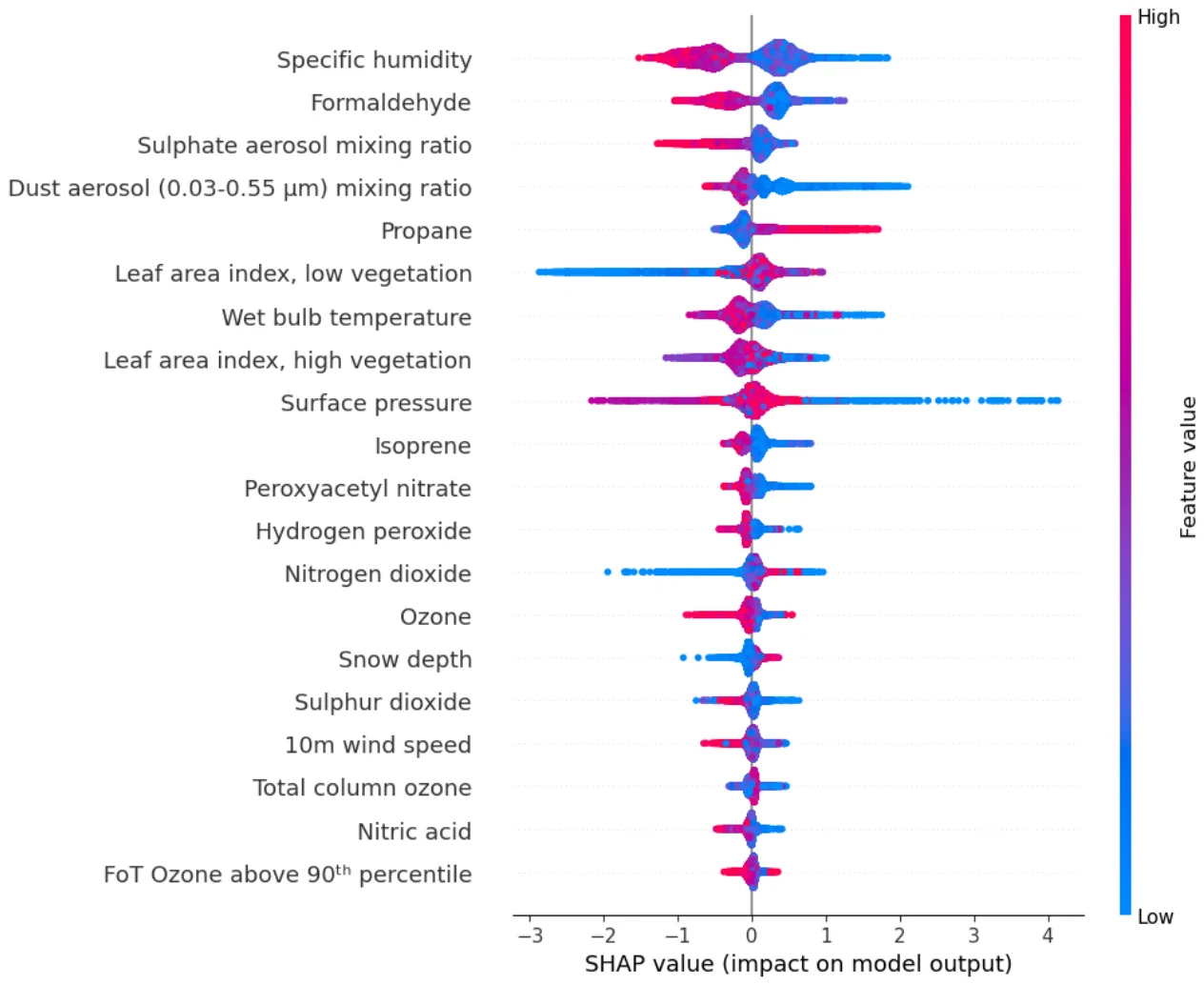
Feature ranking of variables that were most influential in estimating life expectancy for the sub-1-year-old age group according to SHAP values in the training set when 30 features were used for estimating life expectancy. Features are arranged in descending order, with the most influential features at the top. The x-axis is in units of years (unit of life expectancy). Each feature has 41,846 circular dots representing the data points of the training set. Higher feature values are represented in red, and lower feature values are in blue. Data points of each feature having identical SHAP values are stacked on top of each other. Feature values on the left of the central line could possibly decrease the life expectancy, and feature values on the right of the central line could possibly increase the life expectancy.

Figure 8 shows the SHAP values of the 20 top features in the training set that indicate the importance of features in descending order, with the most influential feature at the top. A complete list of features is available at the following link: (https://github.com/mi3nts/Life_Expectancy_Complete/blob/main/Results/shap_30_train.png, accessed on 28 December 2025).

**Figure A3:**
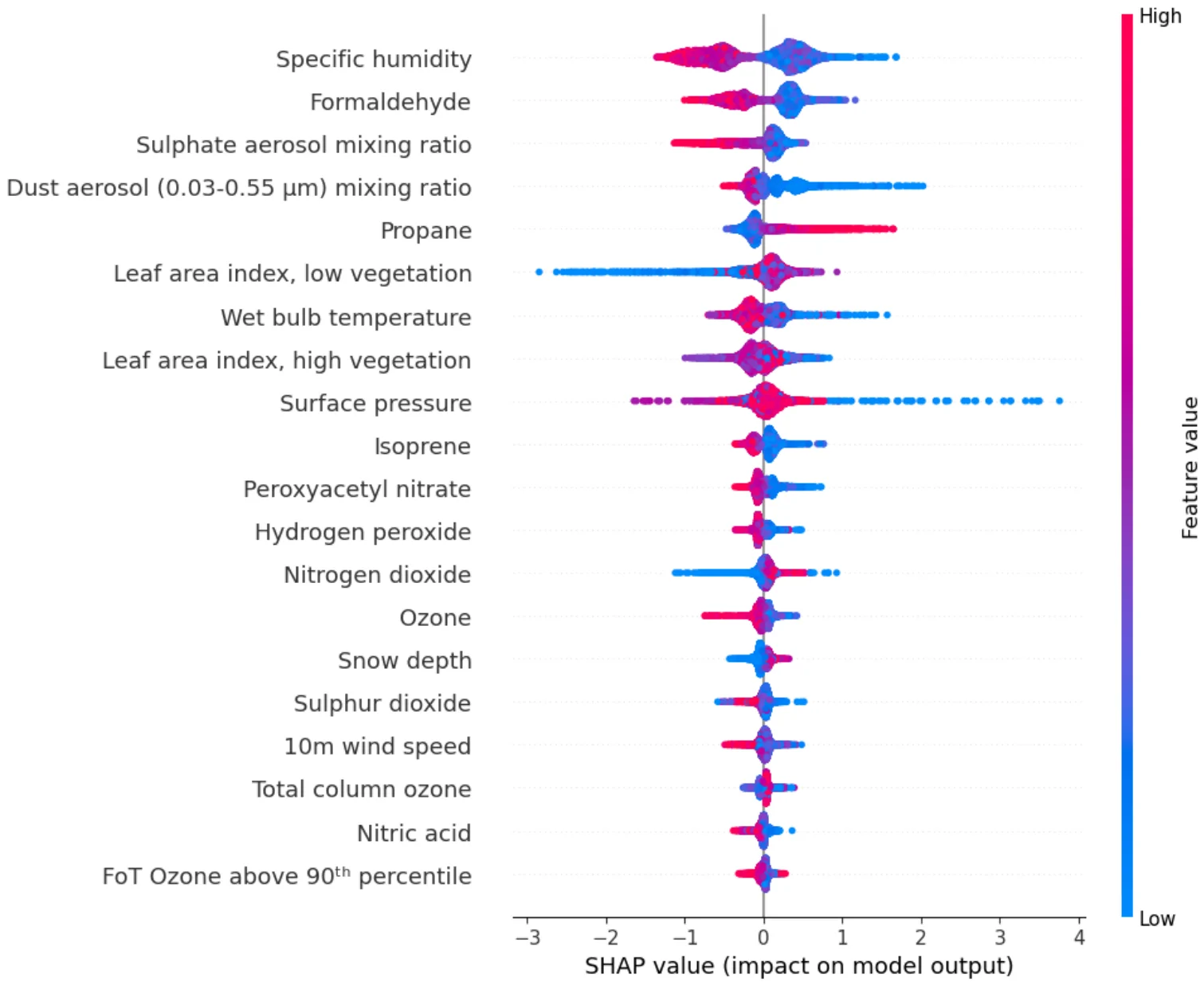
SHAP value beeswarm plot of top 20 features when 30 features were used for estimating life expectancy of sub-1-year-old age group in test set. The X-axis is in the units of year which is the unit of life expectancy. Each of the feature has 10,464 circular dots which is the number of data points in the test set. Figure shows that high values of formaldehyde could possibly decrease the life expectancy as higher values are on the left of the central black line. Similarly, higher values of leaf area index, low vegetation could possibly increase the life expectancy as most of the high feature values are on the right side of the central black line.

The feature ranking in Figure 7 is not consistent with the permutation feature ranking in Figure 8. However, the main variables remain consistent, such as formaldehyde, leaf area index, low vegetation, sulphate aerosol mixing ratio, specific humidity, and wet bulb temperature, indicating the relatively high importance of these variables compared to other variables. These SHAP values also indicate that for pollutants such as formaldehyde, higher values could possibly lower the life expectancy of the sub-1-year-old age group whereas lower values could possibly increase life expectancy of sub-1-year-old age group as higher values of the formaldehyde (indicated in red) are on the left of the central black vertical line, whereas lower values of formaldehyde (indicated in blue) are on the right side of the vertical central black line. Similarly, for the feature leaf area index: low vegetation, a higher index of leaf vegetation (portion of land covered by vegetation of smaller heights) can possibly increase life expectancy of the sub-1-year-old age group, whereas lower values can possibly decrease life expectancy of the same age group. However, a mixture of increase and decrease in life expectancy for sub-1-year-old age group can be seen in the case of leaf area index, high vegetation. As there is a performance difference between the training and testing sets, the SHAP values in the testing set are also calculated. The SHAP values in the testing set for the top 20 features and the entire list are identical to those of the training set. The SHAP values in the testing set in given in Figure A3 in Appendix E.

**Figure 9:**
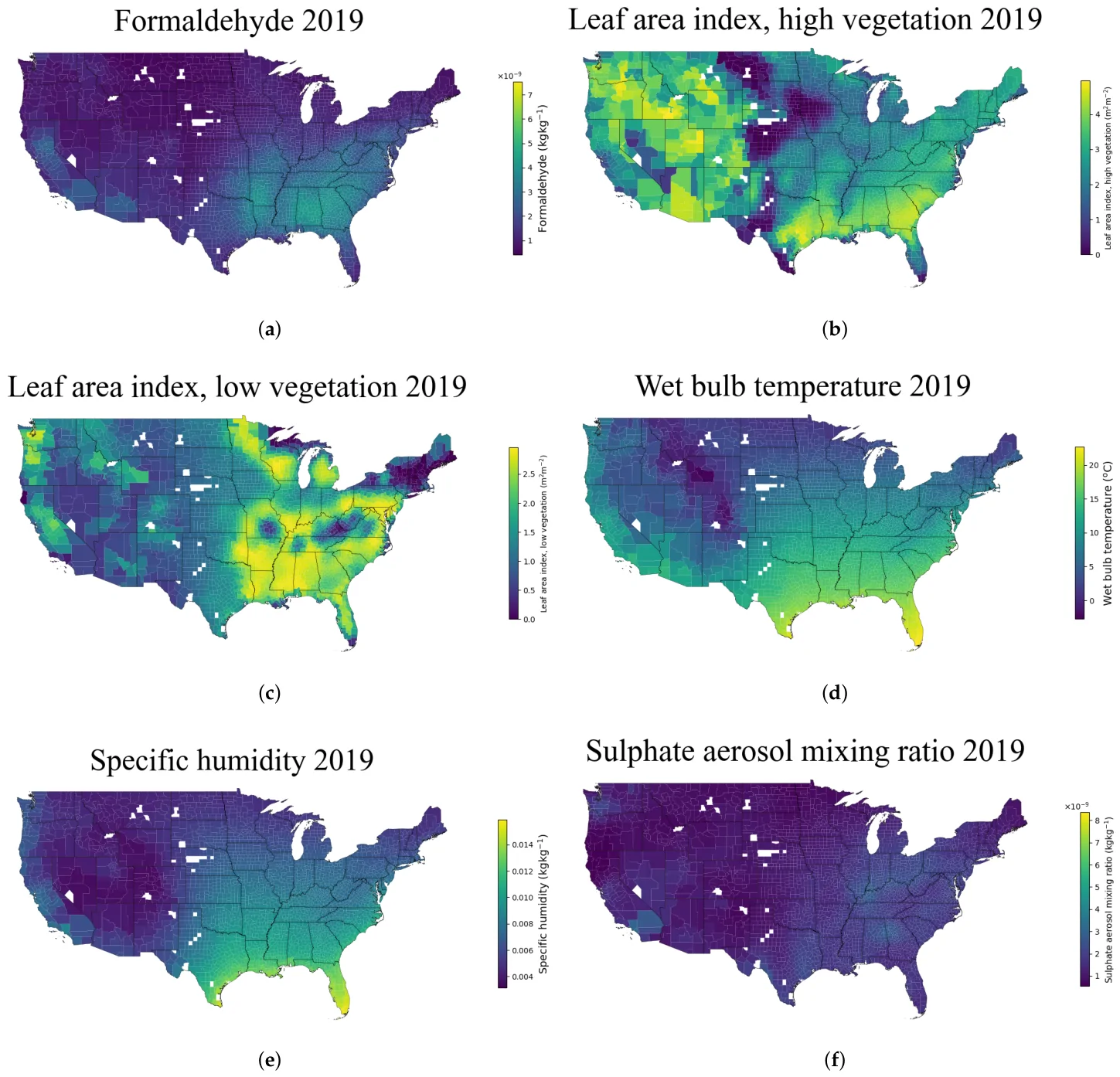
Distribution of (**a**) formaldehyde, (**b**) leaf area index, high vegetation (**c**) leaf area index, low vegetation, (**d**) wet bulb temperature, (**e**) specific humidity, and (**f**) sulphate aerosol mixing ratio in the year 2019 in counties of contiguous USA. White spaces indicate counties where life expectancy data is not available. Formaldehyde, specific humidity, and sulphate aerosol mixing ratio are in the units of ${\mathrm{kgkg}}^{-1}$ which indicates a mass mixing ratio. It is the amount of formaldehyde (or sulphate aerosol mixing ratio or mass of water vapor in case of specific humidity) in a mixture compared to the total amount of all other variables. ${\mathrm{kgkg}}^{-1}$ of formaldehyde and sulphate aerosol mixing ratio can be converted to ppm by multiplying the value in ${\mathrm{kgkg}}^{-1}$ by $10^{6}$. Leaf area index, low vegetation refers to vegetation of small height such as short and tall grass, evergreen shrubs. Leaf area index, high vegetation refers to vegetation of tall heights such as evergreen trees, deciduous trees. Higher the index of these vegetation, higher the portion of the land covered by these vegetation. An index of 0 indicates barren land.

Figure 9 shows the distribution of six variables in the year 2019.

A gif file of these variables from the year 2003 to 2019 is available at the following link (https://github.com/mi3nts/Life_Expectancy_Complete/tree/main/create_gifs, accessed on 28 December 2025) in the corresponding folders having the extension .gif.

Figure 9a shows the high concentration of formaldehyde in the counties of southern regions and in California. As leaf area such as shrubs and trees tends to rarely change throughout the years, changes in leaf area index of both high and low vegetation throughout the years are small. The wet bulb temperature and specific humidity also seem to be high in the southern region of the contiguous US throughout the years. Only a small number of counties have a higher concentration of sulphate aerosols compared to other counties, but it was an important factor affecting life expectancy.

#### 3.2.2. Using Reduced Number of Features

In this section, we estimate the life expectancy of the sub-1-year-old age group using a reduced number of features identified by permutation feature importance, the ranking of which is almost similar to the ranking by SHAP values. We use the top 15, 10, 5, and 3 features from the permutation feature importance as shown in Figure 7 and estimate the life expectancy of the sub-1-year-old age group using the Random Forest algorithm from scikit-learn with optimized hyperparameters. The hyperparameters that are optimized and the ones selected are given in Table A2 in Appendix D.

A summary of the results of estimating life expectancy is given in Table 7.

Table 7 shows that instead of using 30 sets of features, we can use a set of just 5 features to better estimate the life expectancy of the sub-1-year-old age group. The set of 5 features include: specific humidity; leaf area index, low vegetation; leaf area index, high vegetation; sulphate aerosol mixing ratio and wet bulb temperature.

**Figure 10:**
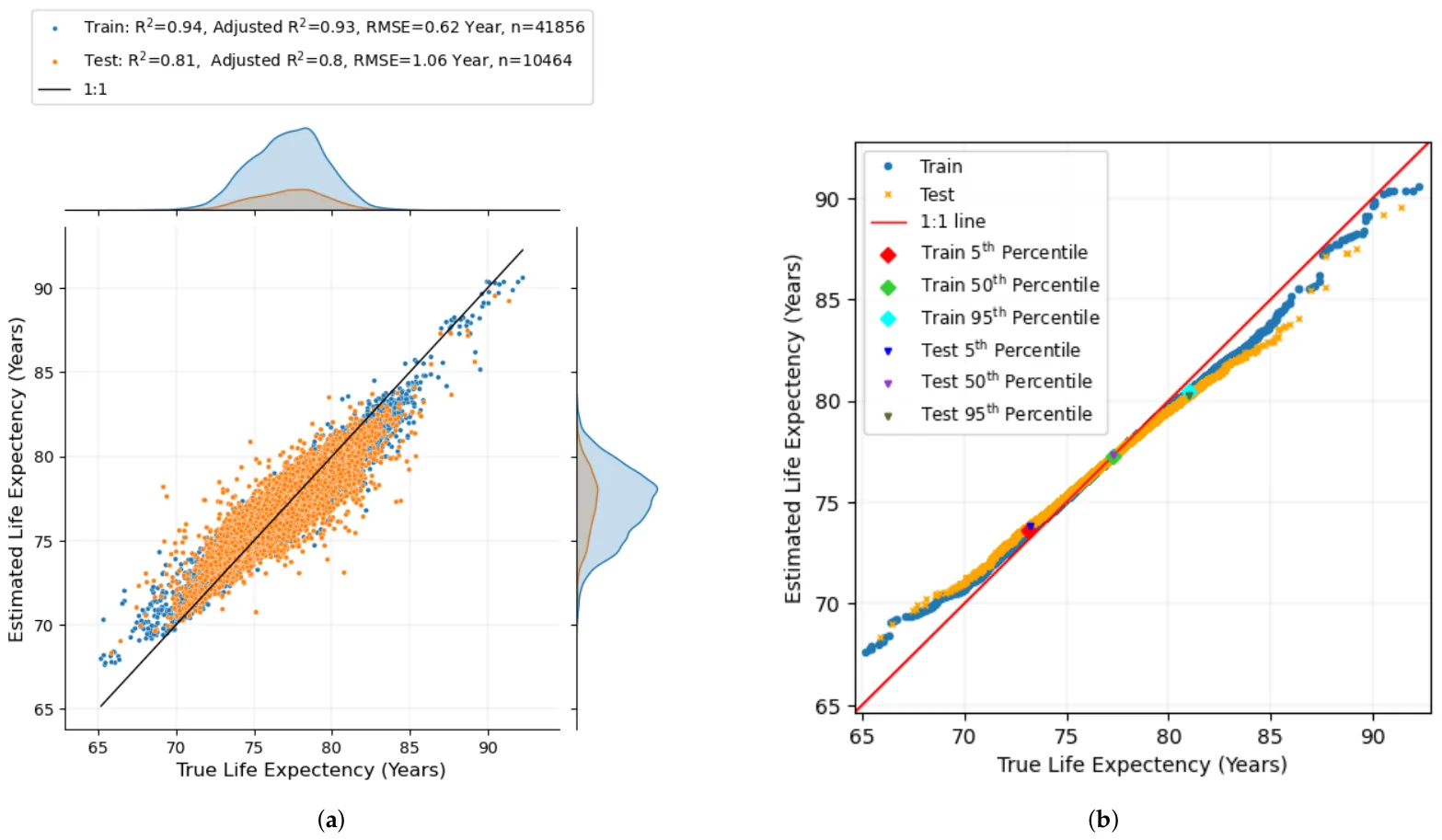
(**a**) Scatter plot of true values of life expectancy of the sub-1-year-old age group plotted against the estimated values of life expectancy of the sub-1-year-old age group when five features were used for estimating the life expectancy of the same age group. A perfect fit is indicated by the black 1:1 line. (**b**) Quantile–quantile plot of true values of life expectancy for the sub-1-year-old age group plotted against the estimated values of life expectancy for the sub-1-year-old age group when five features were used for estimating the life expectancy of the same age group. A perfect fit is indicated by the red 1:1 line. The 5th, 50th, and 95th percentiles in the training and the testing set are also overlaid.

A scatter plot and quantile–quantile plot when five features are used for estimating life expectancy of sub-1-year-old age group are shown in Figure 10. The structure of the scatter plot and quantile–quantile plot is similar to Figure 5a and Figure 5b, respectively. As there are improvements in the estimation of life expectancy when 5 features are used, the data points are closer to their respective 1:1 line compared to when 30 features are used.

## 4. Discussion

Using life expectancy data for the sub-1-year-old age group as provided by IHME, we achieve our first objective, as we have shown the enormous disparity in the life expectancy of the sub-1-year-old age group in the contiguous US. As shown in Table 6, the difference between the highest and lowest life expectancy of the sub-1-year-old age group was greater than 20 years from the years 2003 through 2019. More information on the disparity in life expectancy for the same age group can be found in Figure 4 and the gif of life expectancy for the sub-1-year-old age group from 2003 through 2019, where counties in southern states have relatively lower life expectancy compared to most counties in northern states. Figure 6 which shows the lower 5th and upper 95th percentile also augments the finding as the upper 95th percentile in life expectancy of sub-1-year-old age group was scattered around the US whereas the lower 5th percentile was mostly concentrated in the lower counties of the US.

Our study also shows that we can obtain moderate accuracy in estimating life expectancy of the sub-1-year-old age group in the US using a series of environmental variables. As summarized in Table 7, instead of 30 features, we can use a set of 5 features to achieve even better accuracy in estimating the life expectancy of the sub-1-year-old age group. As 5 features provide better results, the adjusted $R^{2}$ in the test set is higher compared to when 30 features are used, indicating that a small number of features are sufficient. This aligns with Occam’s razor principle, which states that simpler models usually generalize well. Furthermore, this also shows that an increase in the number of variables does not necessarily increase the accuracy of the results as well. Our results are likely distorted due to the small number of data points below the 5th percentile and above the 95th percentile, as shown in the quantile–quantile plot in Figure 5b and Figure 10b. As machine learning models require a large number of data points to learn from a training set and then to be tested on an independent test set due to the small number of data points in this region, the estimation deviate from the perfect 1:1 line. However, the estimation is nearly perfect between the 5th percentile and the 95th percentile, where data points were abundant.

Our key finding is the feature ranking as shown in Figure 7 and Figure 8. These ranking shows formaldehyde to be one of the top factors affecting life expectancy of the sub-1-year-old age group in the US. Formaldehyde, which is found primarily in manufactured wood products, materials used in building, household products such as paints, glues, preservatives used in some medicines and cosmetics, and also in fertilizers [42], is known to cause several problems in the human body related to the respiratory system, acute poisoning, and irritation [16,17]. Figure 9a and the corresponding gif file also show the higher concentration of formaldehyde in the southern states compared to the northern states. The feature ranking in Figure 8 shows that a land area consisting of low vegetation could possibly increase life expectancy of the sub-1-year-old age group, whereas there seem to be mixed results in the case of a land area consisting of high vegetation. Greenness has benefits related to the reduction in mental health issues and air pollution [19,20]. Figure 9b,c, and the corresponding gif files show that the southern states have large areas of low and high vegetation. This indicates that there are other crucial factors that affect life expectancy in addition to vegetation.

Figure 9d and the corresponding gif file of wet bulb temperature show that counties in southern states have higher wet bulb temperature compared to counties in northern states. Figure 8 shows that higher wet bulb temperature could possibly decrease the life expectancy of the sub-1-year-old age group. The wet bulb temperature, which is an indicator of heat stress if it exceeds 35 °C, can potentially result in hyperthermia in mammals, including humans, as in this condition dissipating heat becomes impossible [43]. In our study, we calculate the one-year average of the wet bulb temperature, where the values are around 25 °C. However, there may be instances where the wet bulb temperature is much higher. Better environmental conditions, especially outdoors, where the wet bulb temperature is low, can possibly improve the life expectancy of the sub-1-year-old age group. The SHAP value plot of specific humidity in Figure 9a shows a mixture of red and blue dots on either side of the central black line, indicating that the life expectancy of the sub-1-year-old age group can vary based on the location. Peroxyacetyl nitrate is also one of the main features affecting life expectancy of sub-1-year-old age group, the acute toxicity is similar to nitrogen dioxide, but higher than ${\mathrm{SO}}_{2}$ [44]. More research is needed on the effect of surface pressure on humans. Although we expected pollutant such as ${\mathrm{PM}}_{2.5}$ to be higher in the feature ranking, we did observe pollutants such as sulphate aerosols, dust aerosols, nitrogen dioxide as shown in Figure 7 and Figure 8.

Our results in estimating life expectancy for the sub-1-year-old age group, in which we obtain a moderate accuracy as indicated by RMSE of 1.06 years and $R^{2}$ of 0.81 in an independent test set using a set of five features, clearly indicate missing variables in our study. Nevertheless, since we obtain an $R^{2}$ of 0.81 in an independent test set, this shows that a moderate portion of the study of life expectancy for the sub-1-year-old age group is explained by environmental variables.

There are several limitations in this study that should be acknowledged. The first is the use of repeated counties over multiple years, which can lead to information leakage. For example: if a county of a particular year has appeared in the training set, the same county but that of a different year can appear in the testing set, which can be problematic, especially considering a target variable such as life expectancy of any age group, which remains close to each other for a number of years. Furthermore, the training data may have a county from a year, say 2019, and the testing data may have data from a year before that, which is not feasible in the real world. Future studies can train on data from specific counties of the first few years and test on totally unseen counties of the remaining years to prevent information leakage. Secondly, throughout the manuscript we consistently mention that a decrease in pollutants such as formaldehyde could possibly increase life expectancy of the sub-1-year-old age group, rather than being definite about it. The reason behind this is the use of SHAP values and permutation feature importance, which are not indicators of the actual causes that affect the life expectancy of the sub-1-year-old age group, but rather factors that were influential in estimating the life expectancy of the sub-1-year-old age group. Future studies can make use of causal analysis to further confirm the result. The third limitation is the concern related to spatial autocorrelation, as counties close to each other can possibly have similar environmental and socio-economic conditions as well. If instances of counties close to each other appear in the training and testing sets, the machine learning model could learn the similarity of these variables rather than learning a general relationship. Spatial dependence thus needs to be taken into account, such as splitting training and testing data geographically.

Life expectancy is affected not only by environmental variables but also by several other factors as well. Studies have shown that life expectancy tends to be longer with higher incomes in the United States [45,46]. Between 2001 and 2014 in the United States, people with higher income tended to live longer and the life expectancy gap between income groups also increased [47]. Disease-related factors such as better cardiovascular health have been associated with longer life expectancy [48]. Education has also been considered a crucial factor in affecting life expectancy, with a decrease in life expectancy among people with limited education [49,50]. This fact presents the fourth limitation of the study: there are potential confounding variables in the study. For example, while the concentration of formaldehyde in southern states was found to be high, as shown in Figure 9a, the economic condition of southern states could possibly play an even more vital role. Future studies will need to include socio-economic variables such as income of people, education rate, poverty rate, and other relevant variables such as access to health care for a comprehensive collection of variables to better estimate life expectancy and identify factors affecting life expectancy in the US to improve the livelihood of people. Our random forest and XGBoost models have provided moderate accuracy in estimating life expectancy for the sub-1-year-old age group; future studies can use advanced machine learning algorithms such as neural networks and Long Short-Term Memory that could possibly improve the accuracy. Recently developed feature selection methods that chooses relevant ones and eliminates redundant features can be used to produce a feature ranking as well [51]. The final limitation in this study is the use of county-wise environmental data to infer conclusions about individual populations. Future studies can use a population-weighted methodology in which counties with small and large populations can be assigned small or large weights for definitive conclusions.

## Figures and Tables

**Table 1:** List of variables downloaded from CAMS.

S.N.	Variable	Unit	S.N.	Variable	Unit
1	10m u-component of wind	${\mathrm{ms}}^{-1}$	17	Hydroxyl radical	${\mathrm{kgkg}}^{-1}$
2	10m v-component of wind	${\mathrm{ms}}^{-1}$	18	Isoprene	${\mathrm{kgkg}}^{-1}$
3	2 m dewpoint temperature	Kelvin	19	Nitric acid	${\mathrm{kgkg}}^{-1}$
4	2 m temperature	Kelvin	20	Nitrogen dioxide	${\mathrm{kgkg}}^{-1}$
5	Mean sea level pressure	Pa	21	Nitrogen monoxide	${\mathrm{kgkg}}^{-1}$
6	${\mathrm{PM}}_{1}$	${\mathrm{kgm}}^{-3}$	22	Ozone	${\mathrm{kgkg}}^{-1}$
7	${\mathrm{PM}}_{2.5}$	${\mathrm{kgm}}^{-3}$	23	Peroxyacetyl nitrate	${\mathrm{kgkg}}^{-1}$
8	${\mathrm{PM}}_{10}$	${\mathrm{kgm}}^{-3}$	24	Propane	${\mathrm{kgkg}}^{-1}$
9	Surface geopotential	$m^{2}s^{-2}$	25	Specific humidity	${\mathrm{kgkg}}^{-1}$
10	Surface pressure	Pa	26	Sulphate aerosol mixing ratio	${\mathrm{kgkg}}^{-1}$
11	Total column ozone	${\mathrm{kgm}}^{-2}$	27	Sulphur dioxide	${\mathrm{kgkg}}^{-1}$
12	Carbon monoxide	${\mathrm{kgkg}}^{-1}$	28	Temperature	Kelvin
13	Dust aerosol (0.03–0.55 µm) mixing ratio	${\mathrm{kgkg}}^{-1}$	29	Leaf area index, high vegetation	$m^{2}m^{-2}$
14	Ethane	${\mathrm{kgkg}}^{-1}$	30	Leaf area index, low vegetation	$m^{2}m^{-2}$
15	Formaldehyde	${\mathrm{kgkg}}^{-1}$	31	Snow albedo	(0–1)
16	Hydrogen peroxide	${\mathrm{kgkg}}^{-1}$	32	Snow depth	meter of water equivalent

**Table 2:** List of sources of data and corresponding temporal and spatial resolution.

Data Source	Temporal Resolution	Spatial Resolution
CAMS EAC4	2003 to 2024, every 3 h	0.75 ° × 0.75 °
CAMS EAC4 monthly averaged	2003 to 2024, 1 month	0.75 ° × 0.75 °
ERA5	1940 to present, every hour ^1^	0.25 ° × 0.25 °
IHME	2000 to 2019, annual	Counties of USA

^1^ Data was downloaded every 3 h because of the limitation on the amount of data that can be downloaded at a single time.

**Table 3:** List of feature engineered variables. Fraction of Time is abbreviated as FoT.

S.N.	Variable	S.N.	Variable
1	FoT formaldehyde above 90th percentile	7	FoT temperature above 90th percentile
2	FoT hydroxyl radical above 90th percentile	8	FoT temperature below 10th percentile
3	FoT isoprene above 90th percentile	9	FoT temperature above 90 °F
4	FoT Peroxyacetyl nitrate above 90th percentile	10	FoT temperature below 0 °C
5	FoT ozone above 90th percentile	11	FoT ${\mathrm{PM}}_{2.5}$ above EPA threshold
6	FoT hydrogen peroxide above 90th percentile	12	FoT ${\mathrm{PM}}_{10}$ above EPA threshold

**Table 4:** List of 9 clusters of variables that are multicollinear, identified from the dendrogram. A total of 24 features.

S.N.	Variable	S.N.	Variable
1	${\mathrm{PM}}_{2.5}$, FoT ${\mathrm{PM}}_{2.5}$ above EPA threshold, ${\mathrm{PM}}_{1}$, ${\mathrm{PM}}_{10}$	6	Surface pressure, Surface geopotential
2	Nitrogen dioxide, Nitrogen monoxide	7	Peroxyacetyl nitrate, FoT Peroxyacetyl nitrate above 90th percentile
3	Specific humidity, 2 m dew point temperature	8	Isoprene, FoT Isoprene above 90th percentile
4	Wet bulb temperature, FoT temperature below 0 °C, FoT temperature below 10th percentile, 2 m temperature, Temperature	9	Formaldehyde, FoT fromaldehyde above 90th percentile
5	Hydrogen peroxide, FoT hydrogen peroxide above 90th percentile, FoT temperature above 90th percentile		

**Table 5:** List of remaining 21 features that are not linearly correlated with any other feature.

S.N.	Variable	S.N.	Variable
1	Carbon monoxide	15	Mean sea level pressure
2	Ethane	16	Leaf area index, high vegetation
3	Propane	17	FoT ${\mathrm{PM}}_{10}$ above EPA threshold
4	Nitric acid	18	Hydroxyl radical
5	Sulphate aerosol mixing ratio	19	FoT hydroxyl radical above 90th percentile
6	Sulphur dioxide	20	Relative humidity
7	Snow depth	21	FoT temperature above 90 °F
8	Snow albedo		
9	Total column ozone		
10	Dust aerosol (0.03–0.55 µm) mixing ratio		
11	10 m wind speed		
12	Leaf area index, low vegetation		
13	Ozone		
14	FoT ozone above 90th percentile		

**Table 6:** Disparity in life expectancy of the sub-1-year-old age group as indicated by the highest, lowest, mean, median, and standard deviation from the years 2003 through 2019. The highest life expectancy is between two counties, both of which are located in Colorado, and the county with the lowest life expectancy in each year is Oglala Lakota County in South Dakota.

Year	Highest Life Expectancy (Year)	County	Lowest Life Expectancy (Year)	Mean (Year)	Median (Year)	Standard Deviation (Year)
2003	87.0	Pitkin County	65.4	76.3	76.4	2.2
2004	87.6	Pitkin County	65.7	76.1	76.8	2.2
2005	87.6	Summit County	65.8	76.6	76.7	2.3
2006	88.2	Pitkin County	65.8	76.8	77.0	2.3
2007	88.7	Summit County	66.0	77.0	77.2	2.3
2008	88.8	Summit County	66.4	77.0	77.2	2.3
2009	89.5	Summit County	66.3	77.3	77.5	2.4
2010	89.6	Summit County	66.6	77.5	77.7	2.3
2011	89.4	Summit County	66.5	77.5	77.6	2.4
2012	89.7	Summit County	66.6	77.5	77.6	2.4
2013	89.9	Summit County	66.2	77.4	77.6	2.4
2014	90.4	Summit County	66.0	77.5	77.6	2.5
2015	90.0	Summit County	65.9	77.3	77.4	2.5
2016	90.7	Summit County	65.3	77.1	77.3	2.6
2017	91.0	Summit County	65.3	77.1	77.2	2.6
2018	91.6	Summit County	65.1	77.2	77.3	2.6
2019	92.2	Summit County	65.4	77.4	77.5	2.6

**Table 7:** Summary of results of estimating life expectancy using 30 features and reduced number of features.

Number of Features	Train RMSE (Year)	Test RMSE (Year)	Train $R^{2}$	Test $R^{2}$	Train Adjusted $R^{2}$	Test ${\mathrm{Adjusted R}}^{2}$
30	0.64	1.25	0.93	0.73	0.92	0.72
15	0.64	1.22	0.93	0.75	0.92	0.74
10	0.64	1.17	0.93	0.77	0.92	0.76
5	0.62	1.06	0.94	0.81	0.93	0.80
3	0.71	1.18	0.92	0.77	0.91	0.76

**Figure A1:**
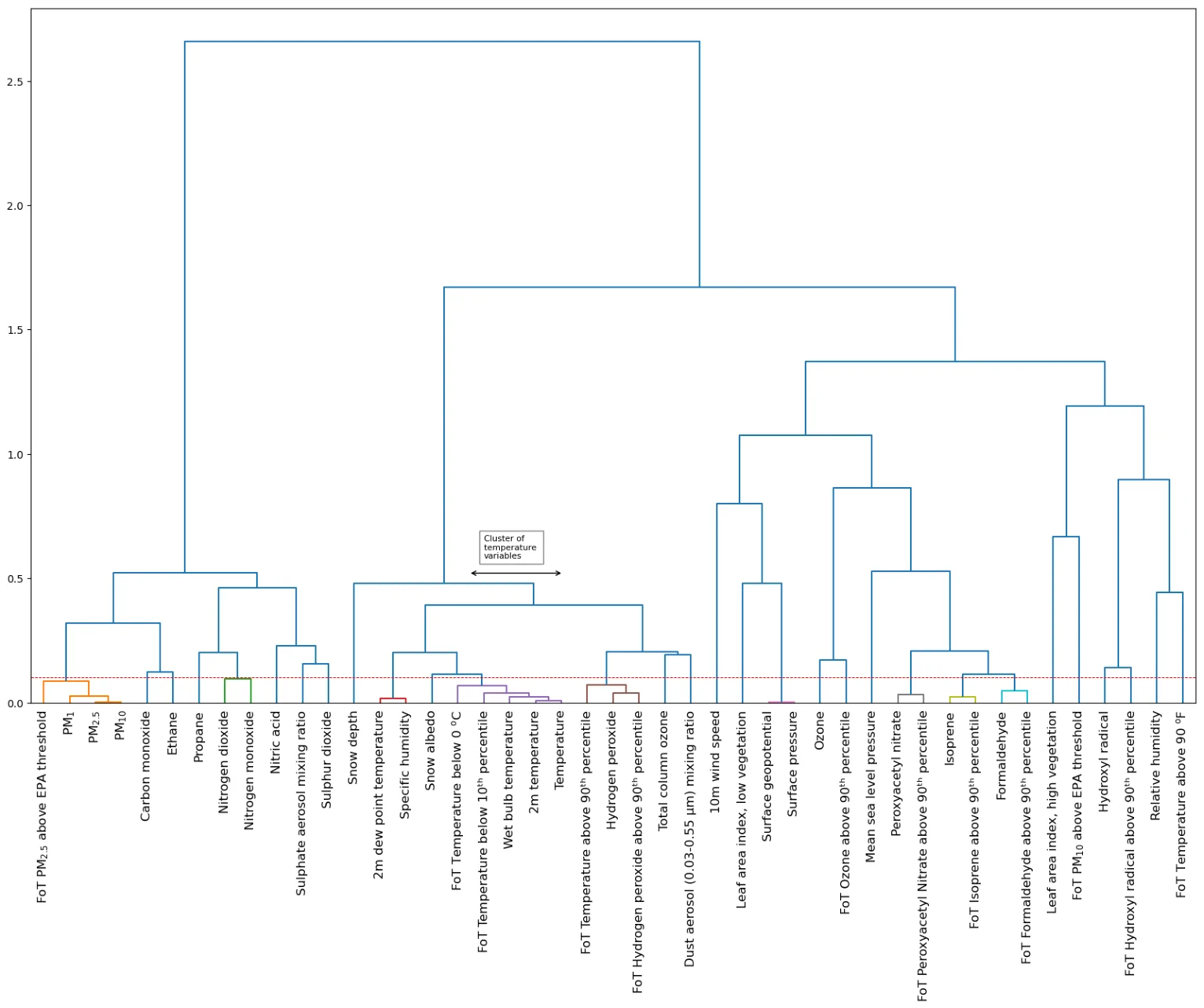
A dendrogram of 45 features to identify multicollinear features. Vertical axis represents the similarities between the variables where smaller distance indicate high similarities. Each unique color below the threshold of 0.10 indicated by the horizontal red line indicates group of multicolinear features. A cluster of temperature variables is also annotated.

## Data Availability

The code to produce the results, figures, and the final dataframe consisting the environmental variables and the target variable is available in GitHub (https://github.com/mi3nts/Life_Expectancy_Complete, accessed on 20 April 2026). The entire project including the environmental data from ECMWF is in Zenodo (https://zenodo.org/records/23199935, accessed on 6 October 2026).

## Abbreviations

The following abbreviations are used in this manuscript:

IHME	Institute for Health Metrics and Evaluation
ECMWF	European Centre for Medium-Range Forecasts
$R^{2}$	Coefficient of Determination
RMSE	Root Mean Square Error
PM	Particulate Matter
GHDx	Global Health Data Exchange
CAMS	Copernicus Atmpshpere Monitoring Service
C3S	Climate Change Service
EAC4	ECMWF Atmoshperic Composition Reanalysis 4
ERA5	ECMWF Reanalysis version5
EPA	Environmental Protection Agency
FoT	Fraction of Time
SHAP	Shapley additive explanations

## Appendix A

**Table A1:** Year and the corresponding number of counties in contiguous USA as available from the shapefiles by United States Census Bureau. Among all the counties for each year, the smallest number of latitude, longitude pair available from the shapefiles and the number of counties with less than 100 pair of latitude and longitude pair is also listed.

Year	Total Number of Counties	Smallest Number of Latitude, Longitude Pair	Number of Counties With Less Than 100 Pair of Latitude And Longitude
2008	3109	129	0
2009	3109	129	0
2010	3109	18	796
2011	3109	129	0
2012	3109	129	0
2013	3109	16	830
2014	3108	14	770
2015	3108	16	772
2016	3108	18	781
2017	3108	14	779
2018	3108	14	784
2019	3108	14	794

## Appendix B

A dendrogram is used to identify multicollinear features. A dendrogram shows hierarchical clustering (the number of clusters is not predetermined) on the Spearman rank-order correlations. From the dendrogram, we manually pick a threshold to group features that are multicollinear and we use a single feature among the group of features. A dengrogram to identify multicollinear features is shown in Figure A1.

The vertical axis of the dendrogram represents the similarities between the variables. The shorter the vertical distance, the higher the collinearity between them, whereas the longer distance indicates less collinearity between the features. The threshold for vertical distance we have chosen is 0.10, which is indicated by the horizontal red line in the figure. This threshold was chosen considering the cluster (or group) of temperatures, which is also annotated in the figure. Choosing a higher threshold will include snow depth in the cluster, whereas choosing a lower threshold will include two temperature variables in the cluster. Each of the clusters below this threshold has a unique color, which indicates that these groups of features have high multicollinearity, whereas the rest of the features are not linearly correlated with any other features.

## Appendix C

A general outline of the calculation of permutation feature importance is given below [41]:

- For a model with a dataset, compute the reference score s, such as $R^{2}$ for regression. The target variable is estimated, and $R^{2}$ is calculated between the true and estimated values.
- For each feature j, randomly shuffle the data in that feature K times and compute the score $s_{k,j}$ ($R^{2}$ value) of the model on the new dataset with only one of the features randomly shuffled and the rest of the feature kept the same.
- The feature permutation $i_{j}$ for a feature $f_{j}$ is then calculated as follows:
  (A1) $$i_{j}=s-\frac{1}{K}\sum_{k=1}^{K}s_{k,j}$$

The result is interpreted as follows:

- High positive value of $i_{j}$: This is interpreted as a feature of high importance. This occurs when the shuffling of the data for a feature leads to a worse prediction (lower values of $s_{k,j}$). It is so because the way a data is related to a target should actually matter.
- High negative value of $i_{j}$: This is interpreted as a feature of low importance. In this case, the shuffling has led to a better prediction (high value of $s_{k,j}$). This means that the data do not represent any dependency with respect to the target variable.

## Appendix D

**Table A2:** Set of 4 hyperparameters that were optimized in Random Forest Models when 30, 15, 10, 5 and 3 features were used for estimating life expectancy of sub-1-year-old age group in the U.S. GridSearchCV was used for optimizing the hyperparameters with 4-fold cross-validation. The hyperparameter that was selected is also provided.

Number of Features	n_estimators	max_features	max_depth	min_samples_split	Optimized Hyperparameter
30	200, 300, 350	10, 20	100, 150	10, 20	300, 20, 100, 10
15	200, 300, 350	5, 10	100, 150	10, 20	350, 10, 150, 10
10	200, 300, 350	5, 8	100, 150	10, 20	300, 8, 100, 10
5	200, 300. 350	3, 4	100, 150	10, 20	300, 4, 150, 10
3	200, 300, 350	2, 3	100, 150	10, 20	300, 3, 100, 10

**Table A3:** Result of estimating life expectancy of sub-1-year-old age group in the US using XGBoost with a set of 30 features. Two hyperparameters max_features and max_depth were optimized.

Train RMSE (Year)	Test RMSE (Year)	Train $R^{2}$	Test $R^{2}$	Train Adjusted $R^{2}$	Test Adjusted $R^{2}$
0.68	1.23	0.92	0.74	0.91	0.73

**Table A4:** Result of 10 fold cross-validation in the training set using Random Forest without optimized hyperparamters.

RMSE (Year) in Each Fold	Mean RMSE	Standard Deviation of RMSE
1.27, 1.21, 1.24, 1.23, 1.28, 1.28, 1.25, 1.29, 1.23, 1.25	1.25	0.02

## Appendix E

The result of feature ranking in the test set using permutation feature importance and SHAP value when 30 features were used to estimate life expectancy is shown in Figure A2 and Figure A3 respectively. The complete list of feature ranking using permutation feature importance is in the link (https://github.com/mi3nts/Life_Expectancy_Complete/blob/main/Results/permutation_importance_30_test.png, accessed on 28 December 2025).

Figure A3 shows the 20 main features in descending order of importance using the SHAP values calculated in the test set when 30 features were used to estimate life expectancy. The complete list of feature ranking using SHAP values is available in the link (https://github.com/mi3nts/Life_Expectancy_Complete/blob/main/Results/shap_30_test.png, accessed on 28 December 2025).
